# Added value of 3D fast-field-echo (FRACTURE) sequences for cervical spondylosis diagnosis: a prospective multi-reader non-inferiority study

**DOI:** 10.1186/s13244-025-01997-5

**Published:** 2025-06-03

**Authors:** Qizheng Wang, Xiaoying Xing, Zixian Zhang, Xiaoxi Ji, Shipei He, Yuxin Yang, Jiajia Xu, Qiang Zhao, Ning Lang

**Affiliations:** 1 Department of Radiology, Peking University Third Hospital, Beijing, PR China; 2 United Imaging Research Institute of Intelligent Imaging, Beijing, PR China; 3 Department of Radiology, State Key Laboratory of Vascular Homeostasis and Remodeling, Peking Third Hospital, Beijing, PR China

**Keywords:** Cervical spondylosis, MRI, CT like image, Diagnosis, Spine

## Abstract

**Objectives:**

To assess the potential of fast field echo resembling a CT using restricted echo-spacing (FRACTURE) sequence to enhance conventional MRI in detecting bone abnormalities of cervical spondylosis.

**Materials and methods:**

137 consecutive patients with cervical spondylosis who underwent clinically indicated paired CT and MRI within 2 weeks between January and June 2024. After routine MRI, the 3D-FRACTURE sequences were performed. Three radiologists independently evaluated the data during three sessions: (1) CT with consensus, (2) routine MRI, and (3) FRACTURE, with a 4-week interval between sessions. Assessments included osteophytes, bony foraminal stenosis, posterior longitudinal ligament ossification (OPLL), their anatomical location, and diagnostic confidence, using CT as the reference standard. Inter- and intra-reader reproducibility was assessed using multi-rater Fleiss κ and the intraclass correlation coefficient (ICC), respectively. The non-inferiority assessment compared routine MRI/FRACTURE and CT diagnoses using a relative reduction margin of 0.5.

**Results:**

The study sample comprised 82 males and 55 females (age 56.9 ± 9.8 years). ICC indicated good to excellent inter-rater reliability for FRACTURE (osteophytes: ICC, 0.83–1.00; OPLL: ICC, 0.73–0.92; bony foraminal stenosis: ICC, 0.76–0.98), which was superior to conventional MRI (most ICC values < 0.7). The diagnostic confidence by FRACTURE sequences was significantly higher than by routine MRI (*p* < 0.001). Non-inferiority analysis demonstrated that FRACTURE and CT detection were similar for osteophyte, bony foraminal stenosis, and OPLL within a margin of 0.5.

**Conclusion:**

The FRACTURE sequence demonstrated comparable performance to CT in bone abnormalities detection in cervical spondylosis, superior to the routine MRI protocol.

**Critical relevance statement:**

The FRACTURE sequence addresses the limitations of conventional MRI in evaluating bone abnormalities, potentially minimizing radiation exposure and streamlining the diagnostic process for patients.

**Key Points:**

- MRI has advantages in the evaluation of cervical spondylosis, but is still insufficient in bone abnormalities evaluation.
- The FRACTURE sequence performed comparably to CT in bone abnormalities detection in cervical spondylosis.
- MRI with FRACTURE sequences may provide a non-ionizing method for assessing cervical spondylosis in some clinical settings.

**Graphical Abstract:**

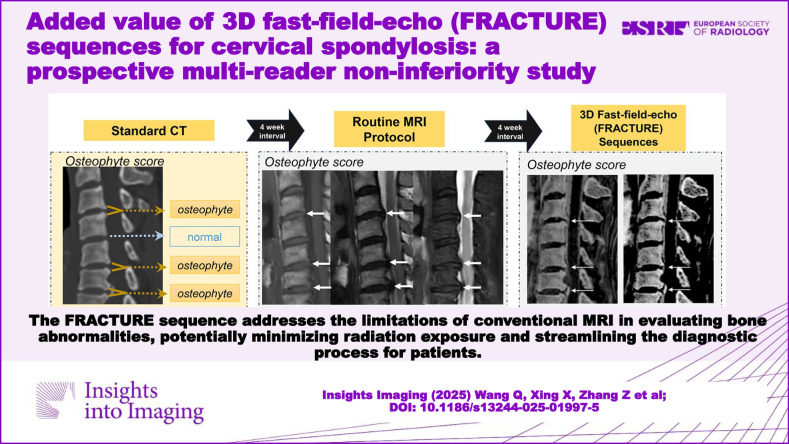

## Introduction

Cervical spondylosis is relatively common, especially among those aged over 55 years, and results in considerable morbidity and disability [1, 2]. It is usually caused by disc herniation, osteophyte bone spurs, and posterior longitudinal ligament ossification (OPLL), resulting in varying degrees of compression of the cervical spinal cord or nerve roots. Missed diagnosis or misdiagnosis could lead to delayed treatment or unnecessary intervention, affecting the prognosis [3]. Therefore, establishing a timely diagnosis is critical to patient outcomes.

Imaging techniques provide excellent anatomical evaluation of the cervical spine with specific benefits and limitations [4]. Radiographs are still the basis, providing an initial understanding. MRI provides better soft tissue resolution, making it possible to include disc/annulus fibrosus, bone marrow, and the spinal cord [5]. However, MRI has apparent bone structure assessment limitations compared to CT [6, 7]. CT scans can characterize bone components well and have contributed to the evaluation of osteophytes, ligament ossification, and bony foraminal stenosis in cervical spondylosis. Because these modalities differ in their advantages, patients with cervical spondylosis are usually examined with all of them to obtain a comprehensive assessment, resulting in increased time, financial costs, and radiation exposure.

MRI with improved bone delineation could complement conventional MRI protocols in evaluating the cervical spine, while reducing ionizing radiation exposure compared to CT. Fast field echo resembling a CT using restricted echo-spacing (FRACTURE) is a new method for observing and evaluating bone structural lesions [8, 9]. FRACTURE is a 3D acquisition sequence with high image resolution that reduces water-lipid chemical shift and motion artifacts by multiple gradient echo acquisitions and subsequent image summation [10]. The bone features are highlighted, and the image signal-to-noise ratio is improved. The image can be reconstructed at any angle conducive to lesion display and diagnosis [11]. The FRACTURE sequence has been preliminarily explored in previous studies, suggesting the potential feasibility of applying it to osseous cervical spine assessment [12]. However, the small sample (only 27 cases) and not in vivo scans (autopsy cases) limited the strength of their conclusions.

By explicitly comparing the imaging quality and diagnostic accuracy of the FRACTURE sequence with CT, we hypothesized that this approach could achieve comparable diagnostic performance while reducing radiation exposure, thereby improving bone structure evaluation on MRI and expanding its clinical applicability.

## Materials and methods

### Patients

**Fig. 1:**
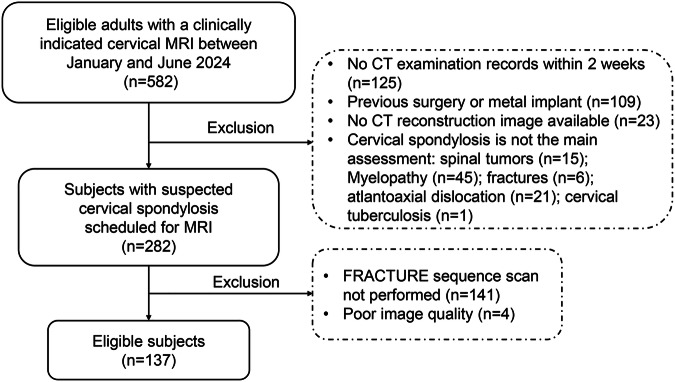
Study flowchart

This prospective study was approved by the institutional review board (M2024079), and written informed consent was obtained from all subjects. Eligible adults with a clinically indicated cervical spine MRI between January and June 2024 were included prospectively. The exclusion criteria included: No CT examination records within 2 weeks (*n* = 125); previous surgery or metal implant (*n* = 109); no CT reconstruction image available (*n* = 23); the main assessment is other than cervical spondylosis, including spinal tumors (*n* = 15), myelopathy (*n* = 45), fractures (*n* = 6), atlantoaxial dislocation (*n* = 21), and cervical tuberculosis (*n* = 1). Finally, 137 patients with cervical spondylosis were continuously recruited for the study. The flowchart of patient inclusion and exclusion is shown in Fig. 1.

### MRI acquisition and software processing

All MRI scans were performed using a 3.0-Tesla scanner (uMR 880, United Imaging Healthcare) with a 16-channel head and neck coil. The 3D-FRACTURE sequence has five in-phase echoes, isotropic voxels (voxel size 0.67 × 0.67 × 0.67 mm), and a field of view of 220 × 200 mm. MRI images with contrast like CT images were acquired by multiple echo sequences with constant echo-spacing. The five echo sequences acquired with the same resolution were set with different echo times (2.26, 4.49, 6.71, 8.93, and 11.15 ms). The echo train length was 33, the pixel bandwidth was 650 Hz/pixel, the acquisition matrix was 288, and the flip angle was 15°. A pre-saturation band was added to alleviate the movement artifacts caused by respiration and swallowing. This process was carried out by the MRI technologist based on their experience, adjusting the placement of the pre-saturation band to optimize artifact reduction while maintaining image quality. Details are provided in the Supplementary Material (Part 2). The overall scanning time of the FRACTURE sequence ranged between 3.50 and 4.25 min.

Custom MATLAB R2021b(R) was utilized for multiple echo data analysis to transform the CT-like images in two steps. First, a magnitude summation of all echo amplitudes was performed to improve the signal-to-noise ratio, leveraging the cumulative effect of the five echoes. Image contrast was enhanced as the signal decreased from the first to the fifth echo to maintain a high signal-to-noise ratio. Second, the grayscale of the cumulated images was inverted, resulting in a CT-like contrast of the bone cortex [8].

### CT imaging

The cervical spine CT was performed using a 64-slice scanner (Somatom Definition Flash, Siemens) with open care dose 4D. The parameters were consistent with those of routine scans without adjustment: 120 kVp; tube current of 1000 mAs; slice thickness, 3.0 mm; pitch, 0.6; delay, 2 s; scan time, 4.32 s; rotation time, 0.5 s. Image data were reconstructed using a bone window (H60) and a soft tissue window (H40) with a layer thickness of 1 mm and a field of view of 200 × 200 mm. The CT scans acted as the reference standard for the presence of osteo-degenerative changes, which is widely recognized for its superior spatial resolution and excellent contrast in imaging bone structures [10].

### Multi-reader evaluation process

All data post-processing and collation work is done by an independent technician (Z.Z.). The datasets were anonymized and randomized for assessment. Three radiologists with different expertise levels (X.X., X.J., and Q.W. with 17, 9, and 5 years of experience, respectively) evaluated the same datasets independently. Before the multi-reader evaluation process, a 1-h seminar session was conducted by a specialist in musculoskeletal radiology (N.L.) with 20 years of experience using CT images of 15 patients outside the cohort. Considering that OPLL can be confused with white line artifacts along the posterior vertebral body edge, especially when window width and level settings are inappropriate, this is an important focus of this section, clarified through expert explanation. A detailed distinction with illustrations can be found in Part 3 of the Supplementary Material. The evaluation process consisted of three stages: CT, conventional MRI, and FRACTURE, with a 4-week interval between stages. After evaluating the CT scans, differences between the three readers were resolved by discussion, which further ensured consistent evaluation criteria among the three readers. The location of the abnormality and diagnostic confidence were recorded for each assessment in the MRI and FRACTURE stages.

### Image assessment

**Fig. 2:**
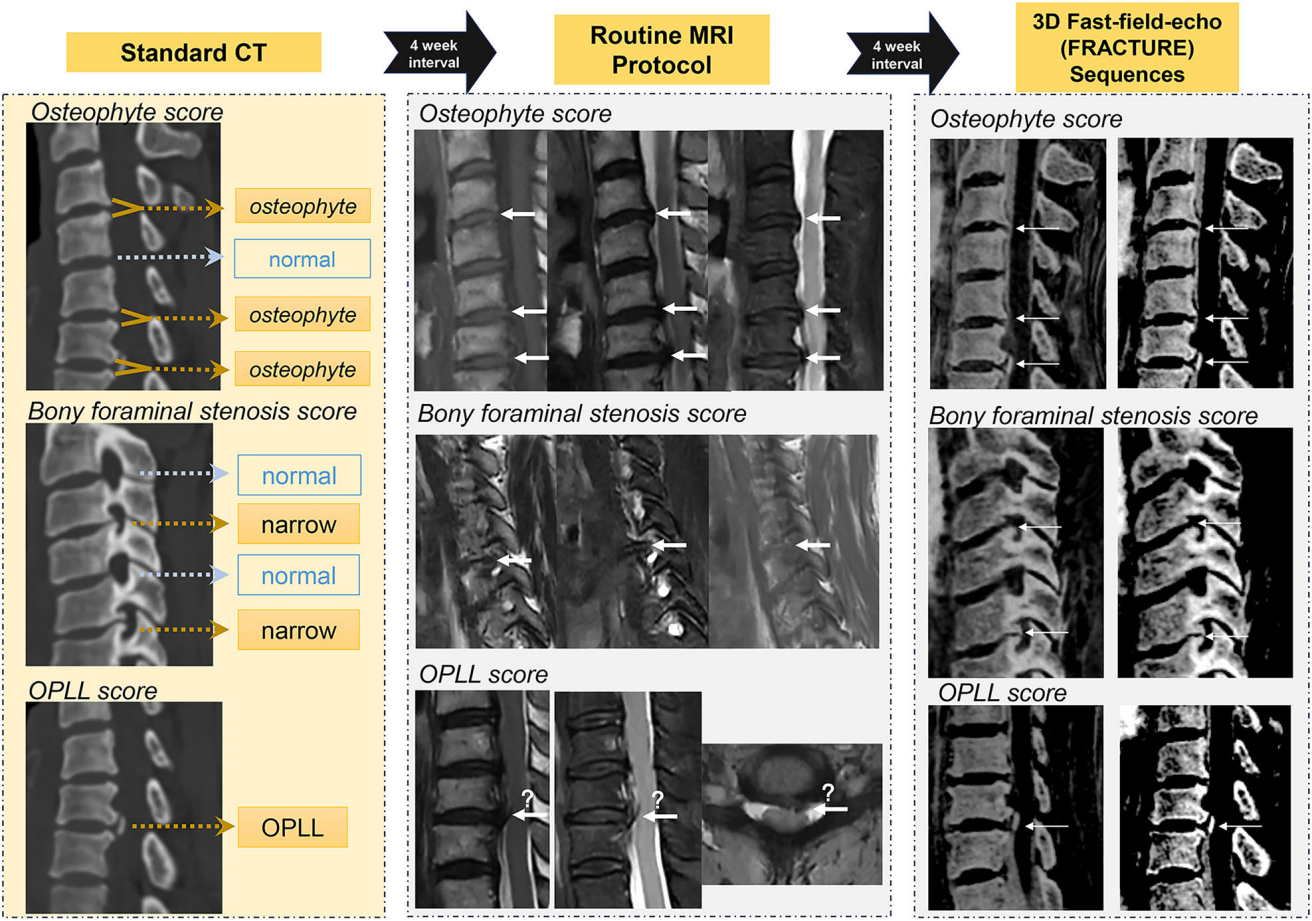
The evaluation process and diagnostic criteria diagram. Three major bone abnormalities of cervical spondylosis were evaluated: osteophytes, bony foraminal stenosis, and posterior longitudinal ligament ossification. The observers were allowed to make routine image adjustments during the evaluation. Two sets of FRACTURE sequences with different window widths and contrasts were presented

The evaluation process and diagnostic criteria are shown in Fig. 2. Each case was evaluated using an anonymous questionnaire concerning osteophytes affecting the central spinal canal, bony foraminal stenosis, and OPLL. The observers could adjust the window width and level settings. Axial and sagittal images could be interactively referenced using position guidelines. The questionnaire focused on the cervical spondylosis bone structure assessed by CT while disregarding aspects clearly shown by routine MRI, such as disc herniation and spinal cord signals.

Detailed evaluation criteria were as follows: (1) Osteophytes involving the central vertebral canal were defined as those on the posterior lower or upper margin of the vertebral body. Equal vertebral signals were shown on conventional T1- and T2-weighted images. Each reader evaluated four intervertebral spaces (C3-4, C4-5, C5-6, and C6-7) and recorded the presence of osteophytes. (2) Bony foraminal stenosis was visually detected based on the shape of the foramina. Normal foramina are oval with smooth bone margins. Positive foramen stenosis was determined when visual changes in the foramen shape at the leading or trailing edge were noted. Advanced osteophytes might lead to occlusion. Stenosis due to disc herniation was not considered. The diagnosis was divided into eight sites (C3-4, C4-5, C5-6, and C6-7 on both sides). (3) OPLL appeared on CT images as a high-density posterior margin of the vertebral body or intervertebral space and was diagnosed at 11 locations (C2, C2-3, C3, C3-4, C4, C4-5, C5, C5-6, C6, C6-7, and C7). The ossified object could be in the center or to one side of the cross-sectional image and could appear as a typical layered structure or an isolated point-like ossification shadow in sagittal images. The OPLL MRI signal is identical to that of the bone cortex.

### Bone abnormality score and diagnostic confidence

All diagnoses were compared to the reference to count the number of true positives. The bone abnormality score was calculated as the number of correct diagnoses at each site. That is, the number of true positive or true negative cases detected by the alternative at each assessment site, with CT results as the gold standard (CT is a perfect score). According to the number of sites mentioned above, the maximum scores for the three bone abnormalities (osteophytes, bony foraminal stenosis, and OPLL) were 4, 8, and 11. If the diagnosis was consistent with CT, the site was counted as one point, and zero if inconsistent. The higher the score of conventional MRI and FRACTURE sequence, the more equivalent to the diagnostic effect of CT, whereas the lower the score, the higher the proportion of false positive/false negative.

The diagnosis confidence was recorded using a 5-point Likert scale (a higher score means greater diagnostic confidence: 1 = least confident, 5 = strongest confidence). The overall diagnostic confidence for each patient was calculated by averaging the diagnostic confidence in all assessed sites with bone abnormalities.

### Statistical analysis

Data was analyzed using MedCalc Statistical Software, Version 20.022 (MedCalc Software Ltd.). The numerical agreement among readers was assessed by the exact Fleiss Kappa, as it assigns greater penalties to larger discrepancies in ordinal ratings [13]. The square-weighted Cohen’s Kappa was used to assess the consistency between the MR protocol and the reference criteria. The reproducibility level was divided into excellent (> 0.9), good (0.7–0.9), moderate (0.5–0.7), and poor (< 0.5). A value of 0.7 was considered the lowest limit for good internal consistency of the test. To determine the distribution of bone abnormalities and diagnostic confidence scores, the Shapiro-Wilk test was performed, revealing non-normal data distribution and necessitating the use of non-parametric tests. The bone abnormality scores were compared using the Wilcoxon signed-rank matched-pairs test. The diagnostic confidence scores were analyzed using the Mann–Whitney *U* test. A significance level (α) of 0.05 was used. The McNemar test was selected to compare the diagnostic performance of routine MRI and FRACTURE sequences, as it is specifically designed for paired binary data. Non-inferiority analysis compared the image evaluation (osteophyte, bony foraminal stenosis, and OPLL scores), treated as continuous variables, to CT (reference standard). Since the score was derived from the number of correct diagnoses, the non-inferiority margin (−Δ) was set to 1, which is generally not expected to significantly impact clinical decision-making. We calculated that a sample of 98 would provide 80% power to detect non-inferiority at a one-sided alpha of 0.05, based on assumed effect size and variability from prior studies. To mitigate potential data loss and enhance the generalizability of our findings, we included additional cases.

## Results

### The demographics of the enrolled participants

The study included 137 participants (82 males and 55 females) who underwent examinations for cervical spondylosis. The mean age was 56.9 ± 9.8 (range 34–79) years. All enrolled participants underwent paired CT, conventional MRI, and FRACTURE sequence scans. Single or multiple-site osteophytes were detected in 40 (29.2%) participants, bony foraminal stenosis in 97 (70.8%), and OPLL in 111 (81.0%).

### Intra- and inter-reader reliability

To evaluate the stability of FRACTURE and conventional MRI in readers of different experience levels, intra- and inter-reader reliability was conducted (Table 1). The agreement between conventional MRI and CT for osteophyte detection ranged between poor and moderate (ICC: 0.15–0.66) in the junior radiologist, while the senior radiologist achieved moderate to good agreement (ICC: 0.60–0.81). In contrast, the FRACTURE sequence demonstrated good to excellent agreement, with higher consistency among readers compared to conventional MRI.

**Table 1:** Inter-observer and inter-modality variability in osteophytes affecting the central spinal canal

	CT				Routine MRI				FRACTURE			
	C3-4	C4-5	C5-6	C6-7	C3-4	C4-5	C5-6	C6-7	C3-4	C4-5	C5-6	C6-7
Observer 1												
Number	6	10	27	17	3	1	12	8	5	9	23	15
*p*-value					0.250	0.022*	0.008*	0.004*	1.00	0.289	1.00	1.00
Observer 2												
Number	6	10	27	17	3	4	8	10	5	9	24	15
*p*-value					0.625	0.180	0.092	0.016*	1.00	1.00	1.00	1.00
Observer 3												
Number	6	10	27	17	3	5	19	12	5	10	24	15
*p*-value					0.250	0.219	0.109	0.063	1.00	-	1.00	1.00
Inter-observer agreement	-	-	-	-	0.90 (0.87, 0.92)	0.53 (0.43, 0.61)	0.84 (0.79, 0.88)	0.86 (0.82, 0.89)	1.00	0.96 (0.95, 0.97)	0.99 (0.98, 0.99)	0.98 (0.97, 0.98)

The 95% confidence interval is presented between parentheses. Numbers represent the number of cases diagnosed as positive. The *p*-values were statistically analyzed by McNemar test

Values in bolded and marked with an asterisk (*) indicate statistically significant differences (*p* < 0.05)

The agreement between conventional MRI and CT for bony foraminal stenosis was insufficiently high, with an ICC of 0.33–0.75 for the junior reader. Additionally, conventional MRI underestimated the number of stenoses compared to CT (*p* < 0.05). Although the agreement among the three readers based on conventional MRI was good (ICC: 0.83–0.96), their consistency with CT was moderate, even for the senior physicians. However, with the FRACTURE sequence, the junior reader’s diagnostic consistency improved to good/excellent levels (ICC: 0.81–0.98), with a significant increase in the number of true positives. Furthermore, the inter-reader agreement using FRACTURE was substantial (ICC: 0.88–0.92), supporting its reliability across different experience levels. Detailed results are shown in Table 2.

**Table 2:** Observer diagnostic performance using routine MRI and FRACTURE sequences for bilateral foraminal bony stenosis with CT as the reference standard

Location		Protocol	Observer 1		Observer 2		Observer 3		ICC**
			ICC*	*p*-value	ICC*	*p*-value	ICC*	*p*-value	
C3-4	Left	P1	0.56	0.06	0.73	1.00	0.75	0.07	0.83
		P2	0.85	1.00	0.76	0.55	0.89	0.38	0.91
	Right	P1	0.58	0.013*	0.77	0.73	0.73	0.34	0.84
		P2	0.84	0.69	0.79	1.00	0.92	0.25	0.89
C3-5	Left	P1	0.53	0.41	0.69	0.80	0.7	0.61	0.87
		P2	0.91	0.06	0.79	1.00	0.92	0.13	0.92
	Right	P1	0.68	0.33	0.76	1.00	0.76	0.58	0.91
		P2	0.81	0.34	0.8	0.58	0.81	0.30	0.96
C3-6	Left	P1	0.75	0.21	0.77	1.00	0.88	0.29	0.92
		P2	0.88	0.73	0.83	1.00	0.92	1.00	0.88
	Right	P1	0.66	0.21	0.75	0.002*	0.77	0.08	0.91
		P2	0.85	0.34	0.76	0.80	0.86	0.18	0.91
C3-7	Left	P1	0.68	0.08	0.77	0.55	0.74	0.09	0.92
		P2	0.8	0.34	0.79	0.39	0.9	0.06	0.92
	Right	P1	0.75	0.77	0.8	1.00	0.81	1.00	0.96
		P2	0.98	1.00	0.9	1.00	0.98	1.00	0.92

The *p*-values were statistically analyzed by McNemar test

*FRACTURE* fast field echo resembling a CT using restricted echo-spacing, *P*_*1*_ conventional MRI, *P*_*2*_ FRACTURE sequence, ICC* intraclass correlation coefficient, ICC** inter-observer agreement

Values in bolded and marked with an asterisk (*) indicate statistically significant differences (*p* < 0.05)

For OPLL detection (Table 3), conventional MRI demonstrated low agreement with CT, with ICC values often below 0.7 and in some cases below 0.5. The FRACTURE sequence showed a clear advantage over conventional MRI, with an improved true positive detection rate and ICC consistently above 0.7, regardless of the reader’s experience level.

**Table 3:** Detailed statistical results for OPLL detection

Location	Protocol	Observer 1		Observer 2		Observer 3		ICC**
		ICC*	*p*-value	ICC*	*p*-value	ICC*	*p*-value	
C2	P_1_	0.75	0.002*	0.75	0.035*	0.75	0.035*	1
	P_2_	0.92	0.42	0.92	0.27	0.92	0.79	1
C2-3	P_1_	1	1	1	0.51	-	0.51	1
	P_2_	1	1	1	1	1	1	1
C3	P_1_	0.51	< 0.0001*	0.62	< 0.0001*	0.62	< 0.0001*	0.93
	P_2_	0.78	0.51	0.8	0.73	0.78	0.34	0.97
C3-4	P_1_	0.72	0.06	0.88	0.15	0.8	0.15	0.96
	P_2_	0.88	1	0.8	1	0.93	0.29	0.97
C4	P_1_	0.55	< 0.0001*	0.58	< 0.0001*	0.59	< 0.0001*	0.97
	P_2_	0.86	1	0.88	1	0.85	0.61	0.98
C4-5	P_1_	0.71	0.001*	0.75	0.007*	0.75	0.002*	0.97
	P_2_	0.79	1	0.79	1	0.84	1	0.96
C5	P_1_	0.45	< 0.0001*	0.49	< 0.0001*	0.5	< 0.0001*	0.95
	P_2_	0.8	0.39	0.8	0.77	0.75	0.58	0.97
C5-6	P_1_	0.67	0.013*	0.73	0.039*	0.75	0.023*	0.95
	P_2_	0.88	1	0.88	1	0.88	1	1
C6	P_1_	0.58	0.001*	0.61	**0**.004*	0.61	0.004*	0.98
	P_2_	0.82	1	0.82	0.77	0.81	1	0.98
C6-7	P_1_	0.62	0.002*	0.69	0.035*	0.66	0.035*	0.96
	P2	0.84	0.42	0.82	0.27	0.84	0.79	0.98
C7	P_1_	0.58	1	0.65	0.51	0.65	0.51	0.95
	P_2_	0.73	1	0.77	1	0.73	1	0.98

The *p*-values were statistically analyzed by McNemar test

*FRACTURE* fast field echo resembling a CT using restricted echo-spacing, *P*_*1*_ conventional MRI, *P*_*2*_ FRACTURE sequence, ICC* intraclass correlation coefficient, ICC** inter-observer agreement

Values in bolded and marked with an asterisk (*) indicate statistically significant differences (*p* < 0.05)

### Comparison of diagnostic performance

Among the three observers, McNemar test found that the diagnostic performance for osteophytes, osseous foraminal stenosis and OPLL using FRACTURE protocol had no significant differences from CT, indicating good classification. However, in the conventional MRI protocol, statistical differences were shown. Conventional MRI has the worst detection efficiency for OPLL, which is statistically different from the reference standard at most of the sites, even for senior observers. For osteophytes and interosseous foramen stenosis, the diagnostic results of observer 1 and observer 2 were statistically different from CT in a few sites. Detailed statistical results are shown in Table 3 and the Supplementary Material (Tables S1–S3).

### Comparison of diagnostic confidence

**Fig. 3:**
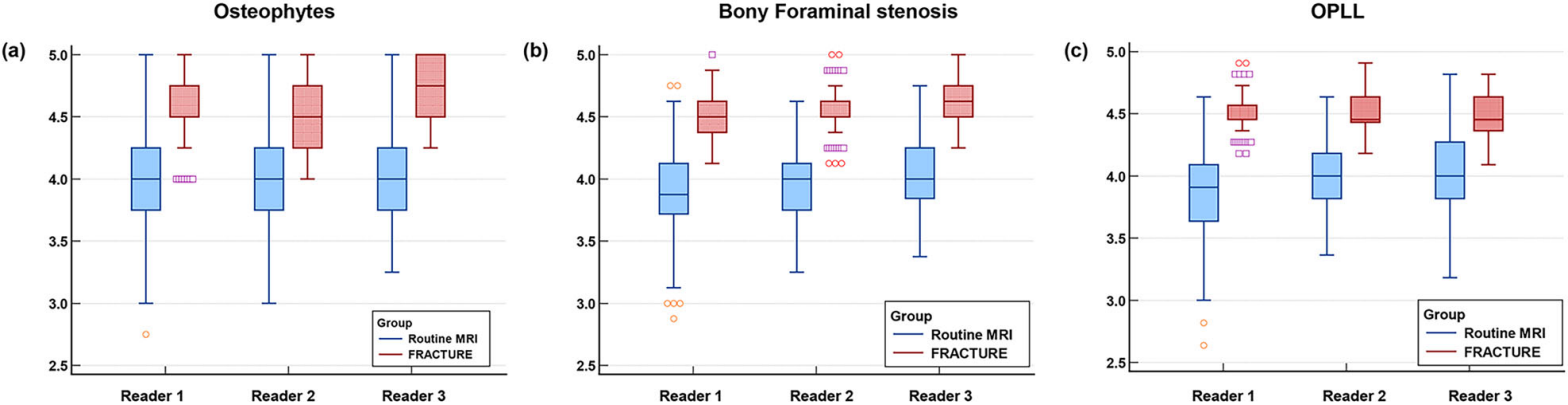
Box plot of diagnostic confidence in conventional MRI protocols and FRACTURE sequences. The two protocols differed significantly in diagnosing bone abnormalities among the three readers (*p* < 0.001). **a** Osteophytes, (**b**) Bony foraminal stenosis, (**c**) OPLL. FRACTURE, fast field echo resembling a CT using restricted echo-spacing; OPLL, ossification of the posterior longitudinal ligament

In addition to diagnostic performance, evaluating diagnostic confidence in different sequences helps to evaluate the reliability and assurance of clinical decision-making. The Likert scores for diagnostic confidence in the FRACTURE images were significantly higher (*p* < 0.001) than when using conventional MRI for diagnosing osteophytes, bony foraminal stenosis and OPLL, regardless of the radiologist’s seniority. Additionally, the interquartile range (IQR) analysis revealed that diagnostic confidence scores were more stable and concentrated in the FRACTURE sequence, whereas scores in the conventional MRI protocol showed greater variability, indicating a less consistent level of confidence. The detailed results are shown in Fig. 3.

### Non-inferiority test of diagnostic performance

**Fig. 4:**
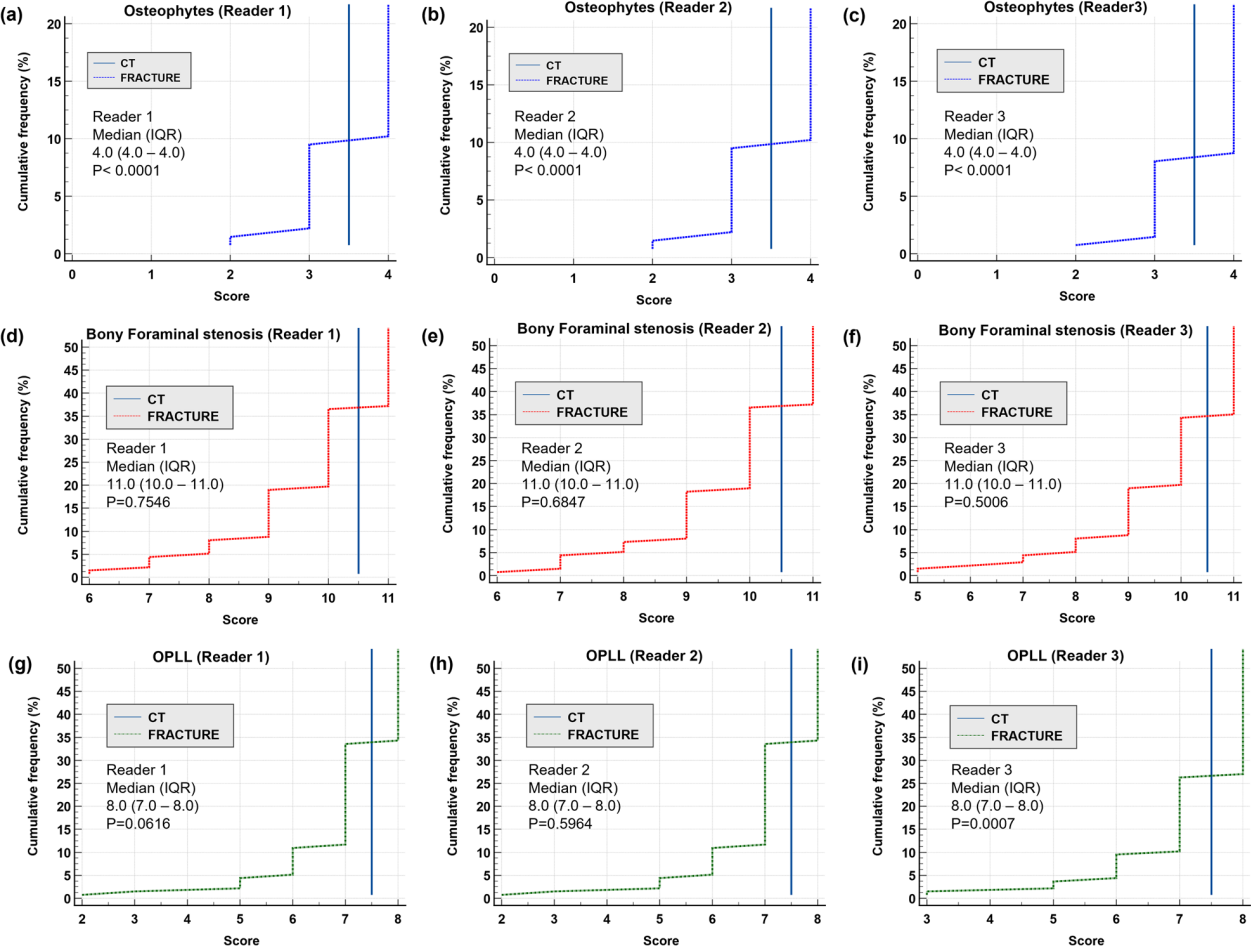
The cumulative frequency distribution graph of the total scores for the accurate detection of bone abnormalities. The blue vertical line indicates score delta = −0.5, using CT (full score) as the reference standard. In the cumulative distribution, the horizontal axis represents the range of values for the score, while the vertical axis represents the percentage of data points that are less than or equal to a particular value. The score difference between the two groups of FRACTURE and CT is shown, and the corresponding confidence intervals and *p*-values are listed. Panels (**a**–**c**) Osteophytes; (**d**–**f**) Bony foraminal stenosis; (**g**–**i**) Ossification of the posterior longitudinal ligament (OPLL), each assessed by Readers 1–3. FRACTUR, fast field echo resembling a CT

**Fig. 5:**
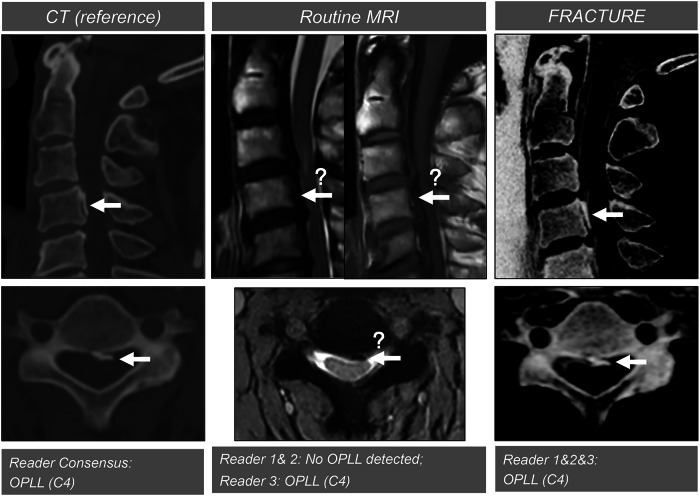
Examples of three imaging protocols for bony cervical spondylosis. From left to right, CT, conventional MRI, and FRACTURE sequences were shown. Conventional MRI sequences underestimated the bony abnormalities, while FRACTURE could be optimized to closely match the CT-diagnosed findings

The difference between the median and the perfect score was less than the predefined non-inferiority margin (Δ = 0.5) for the three readers, indicating that it passed the non-inferiority test. Wilcoxon signed-rank matched-pairs test between the FRACTURE and delta CT (adjusted by subtracting 0.5 from the full score) groups showed that the FRACTURE group did not differ or was better than the control group. Detailed frequency distributions, confidence intervals, and *p*-values are shown in Fig. 4. The FRACTURE is superior (*p* < 0.001) or equal to (*p* > 0.05) the given score difference criteria set for the non-inferiority test. The results of three reader evaluations of the FRACTURE sequence (Fig. 5) show that the cumulative frequency of most scores in the 3 categories of bone abnormalities is distributed on both sides of the critical value and close to the full mark.

## Discussion

Our study revealed that the overall diagnostic performance, diagnostic confidence, inter-method and inter-reader agreements when using the FRACTURE sequence to evaluate cervical spondylosis bone abnormalities, including osteophyte, bony foraminal stenosis, and OPLL, were better than with conventional MRI. The performance of FRACTURE in all reader analyses met the non-inferiority performance goal, demonstrating its potential value in cervical spondylosis assessment.

CT is the modality of choice for skeletal structure of the spine evaluation; however, it utilizes ionizing radiation and has poor soft-tissue contrast [14]. MRI has an incomparable significance in cervical spondylosis evaluation, but its inability to satisfactorily detect bone abnormalities cannot be ignored [15, 16]. It is difficult to accurately delineate bone margins due to relatively long echo times and the limited ability to encode short T2/T2* bone decays [17]. For example, the diagnostic accuracy of OPLL is only 25–60%, as it is difficult to distinguish OPLL from spinal ligament hypertrophy or disc herniation [18]. The low sensitivity of conventional MRI for early ligament calcification or the underestimation of the degree of bone abnormality could affect early diagnosis and the choice of intervention. CT is currently the optimal modality for diagnosing skeletal abnormalities of the spine [19]. While the combination of MRI and CT scans provides comprehensive information, it often requires additional time and financial resources and entails exposure to ionizing radiation. If MRI could provide accurate and detailed visualization of bone lesions and present diagnostic performance equivalent to CT, patients would benefit from cost-saving and a lack of radiation exposure.

Attempts have been made to address the challenge of assessing bone structure details by MRI. MR-generated synthetic CT for the cervical spine has been reported [20, 21], but it was used to assess a single task (OPLL) or some easily observed geometrical analysis (vertebral height, etc.) rather than a comprehensive assessment. Moreover, it faces a greater technical threshold than sequence scanning, and the image authenticity might be questionable for radiologists. To our knowledge, artificial intelligence technologies for multi-task automatic stenosis detection using MRI have not been fully researched.

Studies have shown that it is feasible to use FRACTURE sequences to evaluate bone structures such as the shoulder joint [22, 23], knee joint [24], skull [25], and craniocervical junction [26]. It was mainly used to detect fractures, showing high consistency with CT examinations. FRACTURE achieves high bone contrast by reducing soft tissue contrast through optimized small turnover angles and set echo and repeat times [8]. Moreover, the FRACTURE sequence is more robust to motion artifacts than ultra-short echo time and zero echo time series due to the acquisition of multiple gradient echoes and subsequent image summation [25]. It is based on conventional 3D gradient echo sequences available on most commercial MRI scanners, allowing it to be easily integrated as a supplementary tool in clinical practice. FRACTURE also allows for multi-plane reformatting, such as oblique sagittal reconstruction for the intervertebral foramen and median sagittal reconstruction for the spinal canal. Here, we described our initial experiences with FRACTURE in cervical spondylosis diagnosis, an important routine clinical MRI application with high bone structure evaluation requirements.

We chose to evaluate three aspects of cervical spondylosis, including osteophytes (affecting the central spinal canal), bony foraminal stenosis, and OPLL. Although there might be more underlying bone abnormalities, such as ossification of the ligamentum flavum and the nuchal ligamentum, we excluded them due to low incidence or lack of clinical significance. In the evaluation of inter-method consistency, FRACTURE reached a good to excellent consistency with CT, significantly better than conventional MRI, and was non-inferior to it in the overall evaluation of patients using the three bone abnormality types (osteophyte, κ: 0.83–1.0; foraminal bony stenosis, κ: 0.76–0.98; OPLL, κ: 0.73–0.92). For all parameters, the inter-reader agreement was good to excellent (> 0.8). The high inter-reader agreement indicates that relatively inexperienced radiologists can effectively detect bone abnormalities, which is valuable for primary care units or hospitals with limited resources.

Our results revealed a significant difference between conventional MRI and CT in detecting OPLL, likely due to variations in imaging contrast and anatomical complexity. The presence of subtle abnormalities or indistinct boundaries may further complicate interpretation, underscoring the need for optimized imaging protocols to enhance diagnostic accuracy. FRACTURE sequence showed a stronger consistency with CT, and the difference between the two was not significant (*p* > 0.05), even for junior readers. This means that it is expected to be a promising means to improve MRI detection of OPLL. Integrating FRACTURE sequences into the standard cervical MRI protocol could also enhance diagnostic confidence in evaluating bone abnormalities. However, it is important to note that, despite meeting the non-inferiority testing criteria, it may still underperform in detecting small or complex bone abnormalities due to its lower spatial resolution compared to CT in certain clinical situations.

The FRACTURE sequence (about 4 min) takes longer than CT imaging (< 1 min). While it offers significant advantages, the longer scan time may affect patient throughput and increase the risk of motion artifacts. In our cohort, all scans were acquired on a 3.0-T MRI system without using magnetization preparation pulses, such as inversion recovery or fat suppression. The specific absorption rate remained within safe limits during all scans, providing adequate image quality with a moderate flip angle (~ 15°) and long repetition time, while minimizing radiofrequency energy deposition. However, further large-scale clinical studies are needed to fully validate this. Other inherent limitations of MRI may require clinicians to carefully assess the risks and benefits based on the clinical scenario. For instance, the intense magnetic field may attract metal objects, both internal and external to the body, potentially causing harm or interference with medical devices. Extended scan times and the noise generated during imaging may also contribute to patient discomfort and other operational challenges. To address efficiency concerns, fast imaging techniques such as compressed sensing or deep learning could be used to accelerate FRACURE scans in the future. Additionally, the equipment costs, scanning fees, and maintenance costs of MRI are higher than those of CT, and further research is needed to clarify the cost-benefit ratio through financial aspects.

There are several limitations to this study. First, pathology, as the gold standard cervical spondylosis diagnostic method, is difficult to achieve; in this study, we used CT instead. Second, OPLL thickness assessment was not used because it is often easy to diagnose the thickest site while overlooking sites critical for early diagnosis and treatment. Third, a comparison of objective image quality parameters with conventional sequences was not performed because FRACTURE is a 3D sequence (it lacks comparability). Thus, it is still worth exploring whether the diagnostic differences arise from the protocol’s advantages or variations in image quality. Fourth, cervical curvature, endplate sclerosis, and other such manifestations were not compared. We hypothesized that adding the FRACTURE sequence could improve MRI diagnosis of cervical radiculopathy, OPLL, and other bone details because we believe that X-ray and routine T1WI images are sufficient for evaluation. Fifth, although we implemented a 4-week interval to minimize immediate recall bias, the possibility of memory effects influencing observer evaluations cannot be entirely ruled out. Sixth, this study focused on overall diagnostic performance, and subgroup analyses based on demographic factors were not conducted due to sample size limitations. Last but not least, excluding implant patients may limit applicability, while training, protocol compatibility, and 1.5-T scanner limits may affect clinical use. Future research should consider these factors to better evaluate real-world feasibility and adoption.

## Conclusion

The FRACTURE sequence can help visualize bony structures, including osteophytes, bony foraminal stenosis, and OPLL. It could provide additional information for the diagnosis and treatment of some patients and is expected to complement preoperative CT scans in some clinical scenarios.

## Supplementary information

ELECTRONIC SUPPLEMENTARY MATERIAL

## Data Availability

Data used in this study can be obtained upon request from the corresponding author.

## Abbreviations

FRACTURE: Fast field echo resembling a CT using restricted echo-spacing
ICC: Intraclass correlation coefficient
OPLL: Posterior longitudinal ligament
