# Supplementary material for: Added value of 3D fast-field-echo (FRACTURE) sequences for cervical spondylosis diagnosis: a prospective multi-reader non-inferiority study
Source: Insights Imaging. 2025 Jun 3;16:114. doi: 10.1186/s13244-025-01997-5 (PMC12133650; doi:10.1186/s13244-025-01997-5)
Supplement: Supplementary file 1 — ELECTRONIC SUPPLEMENTARY MATERIAL [file 13244_2025_1997_MOESM1_ESM.pdf]

Added Value of 3D Fast-field-echo (FRACTURE) Sequences for Cervical Spondylosis Diagnosis: A Prospective Multi-Reader Non-Inferiority Study

ELECTRONIC SUPPLEMENTARY MATERIAL

Part 1: Interpretation of the parameters of FRACTURE sequence

Echo Times (2.26, 4.49, 6.71, 8.93, and 11.15 ms): These values were selected to acquire multiple in-phase echoes with constant echo spacing, optimizing signal contrast between bone and surrounding soft tissues. The range of echo times ensures that the combined signal enhances the depiction of bone morphology while suppressing artifacts.

Isotropic Voxel Size (0.67 × 0.67 × 0.67 mm): This resolution was chosen to allow for high-quality multiplanar reconstructions similar to CT, improving spatial resolution for detailed bone assessment.

Field of View (220 × 200 mm): This parameter was set to ensure comprehensive coverage of the cervical spine while maintaining optimal resolution for diagnostic interpretation.

Echo Train Length (33) and Pixel Bandwidth (650 Hz/pixel): These values were optimized to balance scan time, image resolution, and signal-to-noise ratio, reducing distortion and ensuring sharp bone-edge delineation.

Acquisition Matrix (288): This matrix size was chosen to achieve an optimal trade-off between image quality and scan efficiency, allowing for high-resolution imaging within a clinically feasible time frame.

Flip Angle (15°): A lower flip angle was selected to maintain adequate signal intensity while preventing excessive T1 weighting, ensuring clear visualization of bone structures.

Pre-saturation Band: Implemented to mitigate motion artifacts from respiration and swallowing, which are common challenges in cervical spine imaging.

Part 2: Pre-Saturation Band

In cervical spine MRI, a pre-saturation band is strategically placed to reduce motion artifacts caused by respiration and swallowing. These artifacts can degrade image quality and obscure important anatomical details, making it essential to apply pre-saturation techniques effectively. The optimal placement of the pre-saturation band depends on the primary source of artifacts and the imaging sequence being used.

In this MRI scan setup, a pre-saturation band is applied with the following parameters:

- Type: Free pre-saturation band
- Thickness: 40 mm
- Direction: C > T (-9.2°), indicating a tilted orientation from cranial to thoracic
- Position: L: 0.3 mm (left offset), A: 30 mm (anterior offset)
- Special Pre-Saturation Band: Parallel
- Bilateral Application: Enabled for both right (R) and left (L) sides
- Additional Band Thickness: 50 mm
- Spacing Between Bands: 5 mm
- Orientation: Axial placement: Best for suppressing localized signals from the trachea, esophagus, or large blood vessels; Sagittal placement: Applied in cases where pulsation artifacts from the carotid artery interfere with spinal cord imaging.

Part 3 Clarification on differentiating the white line from OPLL

■

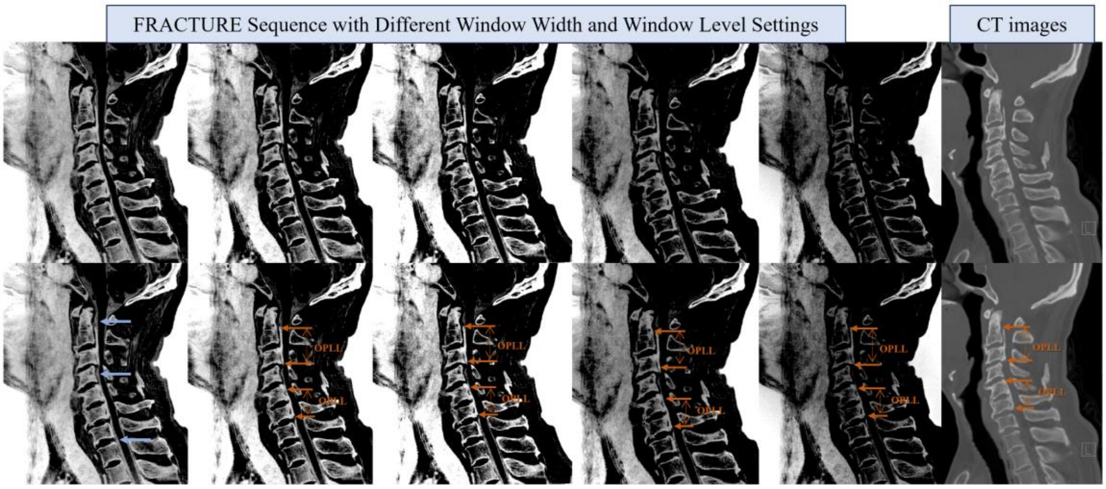

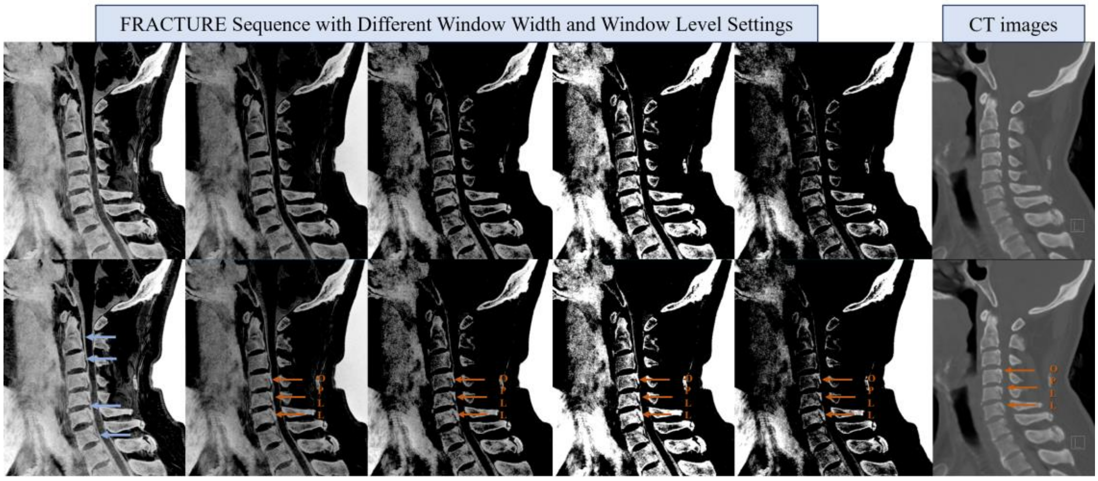

The first row of images presents the original, unannotated images with varying window width and level adjustments, while the second row displays the same images, annotated with blue and red arrows. As observed in the first and second columns, the dura mater appears as a white line with a similar appearance to that of OPLL, making it challenging to visually distinguish between OPLL and the artefact. However, as the window width and level settings are adjusted, the white line associated with the dura mater gradually diminishes and eventually disappears. In contrast, the posterior vertebral body edge typically appears as a faint linear high signal and is separated from the dura mater/OPLL

by a visible gap. When this gap disappears, localized thickening becomes more apparent, allowing for clear differentiation from the dura mater.

- **Blue arrows**: White line (dura mater), thin and uniform in thickness.
- **Red arrows**: OPLL, exhibiting localized thickening.

The window width (W) and level (L) settings, from left to right, are as follows: W900/L3400; W620/L3415; W430/L3500; W440/L3600; W270/L3600.

Part 4: Additional detailed analysis results

Table S1. Inter-observer and inter-modality variability in osteophytes affecting the central spinal canal

	**CT**				**Routine MRI**				**FRACTURE**			
	C3-4	C4-5	C5-6	C6-7	C3-4	C4-5	C5-6	C6-7	C3-4	C4-5	C5-6	C6-7
**Observer 1**												
Number	6	10	27	17	3	1	12	8	5	9	23	15
ICC	-	-	-	-	0.66<br>(0.30, 1.00)	0.15<br>(-0.13, 0.42)	0.50<br>(0.31, 0.69)	0.61<br>(0.38, 0.84)	0.83<br>(0.59, 1.00)	0.94<br>(0.83, 1.00)	0.84<br>(0.72, 0.95)	0.87<br>(0.74, 0.99)
p value					0.250	0.022*	**0.008***	**0.004***	1.00	0.289	1.00	1.00
**Observer 2**												
Number	6	10	27	17	3	4	8	10	5	9	24	15
ICC	-	-	-	-	0.56<br>(0.22, 0.95)	0.19<br>(-0.03, 0.40)	0.36<br>(0.16, 0.57)	0.71<br>(0.52, 0.91)	0.83<br>(0.59- 1.00)	0.94<br>(0.83, 1.00)	0.86<br>(0.75, 0.97)	0.87<br>(0.74, 0.99)
p value					0.625	0.180	0.092	**0.016***	1.00	1.00	1.00	1.00
**Observer 3**												
Number	6	10	27	17	3	5	19	12	5	10	24	15
ICC	-	-	-	-	0.66<br>(0.30, 1.00)	0.60<br>(0.32, 0.89)	0.75<br>(0.60, 0.90)	0.81<br>(0.65, 0.97)	0.83<br>(0.59, 1.00)	1.0	0.86<br>(0.75, 0.97)	0.90<br>(0.78, 1.00)
p value					0.250	0.219	0.109	0.063	1.00	-	1.00	1.00
Inter-observer agreement	-	-	-	-	0.90<br>(0.87, 0.92)	0.53<br>(0.43, 0.61)	0.84<br>(0.79, 0.88)	0.86<br>(0.82, 0.89)	1.00	0.96<br>(0.95, 0.97)	0.99<br>(0.98, 0.99)	0.98<br>(0.97, 0.98)

The 95% confidence interval is presented between parentheses. The intraclass correlation coefficient (ICC) represents the comparison with the CT diagnostic results. Numbers represent the number of cases diagnosed as positive. The *p*-values were statistically analyzed by McNemar test. *****, *p* <0.05.

Table S2. Observer diagnostic performance using routine MRI and FRACTURE sequences for bilateral foraminal bony stenosis with CT as the reference

	**C3-4**						**C4-5**						**C5-6**						**C6-7**					
	**Left**			**Right**			**Left**			**Right**			**Left**			**Right**			**Left**			**Right**		
	**CT**	**P_1**	**P_2**	**CT**	**P_1**	**P_2**	CT	P_1	P_2	CT	P_1	P_2	CT	P_1	P_2	CT	P_1	P_2	CT	P_1	P_2	CT	P_1	P_2
**Observer 1**																								
TP	31	17	27	24	12	20	34	20	34	38	27	31	54	43	49	58	50	51	36	24	29	32	25	31
ICC*	-	0.56	0.85	-	0.58	0.84	-	0.53	0.91	-	0.68	0.81	-	0.75	0.88	-	0.66	0.85	-	0.68	0.8	-	0.75	0.98
95% CI	-	*0.38<br>0.73*	*0.75<br>0.96*	-	*0.38<br>0.77*	*0.72<br>0.97*	-	*0.36<br>0.70*	*0.83<br>0.99*	-	*0.54<br>0.82*	*0.70<br>0.92*	-	*0.64<br>0.86*	*0.79<br>0.96*	-	*0.54<br>0.79*	*0.76<br>0.94*	-	*0.53<br>0.82*	*0.69<br>0.92*	-	*0.62<br>0.88*	*0.94<br>1.00*
p value		.063	1.00		**.013***	.688		.405	.063		.332	0.344		.210	.727		.210	.344		.077	.344		.774	1.0
**Observer 2**																								
TP	31	24	24	24	19	19	34	27	29	38	30	33	54	46	48	58	56	51	36	28	29	32	27	29
ICC*	-	0.73	0.76	-	0.77	0.79	-	0.69	0.79	-	0.76	0.80	-	0.77	0.83	-	0.75	0.76	-	0.77	0.79	-	0.80	0.90
95% CI	-	*0.59<br>0.87*	*0.63<br>0.90*	-	*0.62<br>0.91*	*0.65<br>0.93*	-	*0.55<br>0.83*	*0.67<br>0.91*	-	*0.63<br>0.88*	*0.69<br>0.91*	-	*0.66<br>0.88*	*0.74<br>0.93*	-	*0.65<br>0.86*	*0.65<br>0.87*	-	*0.64<br>0.89*	*0.67<br>0.91*	-	*0.68<br>0.92*	*0.81<br>0.99*
p value		1.00	0.549		.727	1.0		.804	1.0		1.0	.581		1.0	1.0		**.002***	.804		.549	.388		1.0	1.0
**Observer 3**																								
TP	31	22	27	24	17	21	34	25	34	38	30	31	54	48	52	58	54	51	36	26	30	32	27	31
ICC*	-	0.75	0.89	-	0.73	0.92	-	0.70	0.92	-	0.76	0.81	-	0.88	0.92	-	0.77	0.86	-	0.74	0.90	-	0.81	0.98
95% CI	-	*0.61<br>0.89*	*0.80<br>0.98*	-	*0.57<br>0.89*	*0.83<br>1.00*	-	*0.56<br>0.84*	*0.85<br>1.00*	-	*0.63<br>0.88*	*0.70<br>0.92*	-	*0.79<br>0.96*	*0.86<br>0.99*	-	*0.66<br>0.87*	*0.78<br>0.95*	-	*0.61<br>0.87*	*0.81<br>0.99*	-	*0.70<br>0.93*	*0.94<br>1.00*

	C3-4						C4-5						C5-6						C6-7					
p value		.065	0.375		.344	0.250		.607	.125		.581	.302		.289	1.0		.076	.180		.092	.063		1.0	1.0
ICC**	**-**	**0.83**	**0.91**	**-**	**0.84**	**0.89**	-	0.87	0.92	-	0.91	0.96	-	0.92	0.88	-	0.91	0.91	-	0.92	0.92	-	0.96	0.92

standard

Note: P_1, conventional MRI; P_2, FRACTURE sequence; TP, true positive; ICC*, intraclass correlation coefficient; CI, confidence interval; ICC**, inter-observer agreement.

The *p*-values were statistically analyzed by McNemar test. *, p <0.05.

Table S3. Observer diagnostic performance using routine MRI and FRACTURE sequences for ossification of the posterior longitudinal ligament.

	C2			C2-3			C3			C3-4			C4			C4-5			C5			C5-6			C6			C6-7			C7		
	CT	P_1	P_2	CT	P_1	P_2	CT	P_1	P_2	CT	P_1	P_2	CT	P_1	P_2	CT	P_1	P_2	CT	P_1	P_2	CT	P_1	P_2	CT	P_1	P_2	CT	P_1	P_2	CT	P_1	P_2
**Observer 1**																																	
TP	21	14	21	14	14	14	51	28	46	26	20	24	82	52	76	35	24	29	94	55	88	44	28	40	78	48	70	27	15	23	35	18	28
ICC*	-	.75	.92	-	1.0	1.0	-	.51	.78	-	.72	.88	-	.55	.86	-	.71	.79	-	.45	.80	-	.67	.88	-	.58	.82	-	.62	.84	-	.58	.73
95% CI	-	*0.58*<br>*0.91*	*0.83*<br>*1.0*	-	-	-	-	*0.37*<br>*0.66*	*0.68*<br>*0.89*	-	*0.56*<br>*0.87*	*0.78*<br>*0.98*	-	*0.43*<br>*0.68*	*0.78*<br>*0.95*	-	*0.57*<br>*0.85*	*0.67*<br>*0.91*	-	*0.33*<br>*0.58*	*0.69*<br>*0.91*	-	*0.53*<br>*0.81*	*0.80*<br>*0.97*	-	*0.46*<br>*0.70*	*0.73*<br>*0.92*	-	*0.45*<br>*0.80*	*0.72*<br>*0.95*	-	*0.41*<br>*0.74*	*0.60*<br>*0.86*
p value		***.07***	***.25***		***1.0***	***1.0***		***.002****	***.42***		***1.0***	***1.0***		***< .0001****	***.51***		***.06***	***1.0***		***< .0001****	***1.0***		***.001****	***1.0***		***<.0001****	***.39***		***.013****	***1.0***		***.001****	***1.0***
**Observer 2**																																	
TP	21	14	21	14	14	14	51	34	47	26	23	24	82	54	77	35	26	29	94	58	88	44	31	40	78	50	71	27	17	23	35	21	30
ICC*	-	.75	.92	-	1.0	1.0	-	.62	.80	-	.88	.80	-	.58	.88	-	.75	.79	-	.49	.80	-	.73	.88	-	.61	.82	-	.69	.82	-	.65	.77
95% CI	-	*0.58*<br>*0.91*	*0.83*<br>*1.00*	-	-	-	-	*0.49*<br>*0.76*	*0.70*<br>*0.90*	-	*0.78*<br>*0.98*	*0.67*<br>*0.92*	-	*0.45*<br>*0.70*	*0.80*<br>*0.96*	-	*0.63*<br>*0.89*	*0.67*<br>*0.91*	-	*0.36*<br>*0.61*	*0.69*<br>*0.91*	-	*0.61*<br>*0.86*	*0.80*<br>*0.97*	-	*0.49*<br>*0.73*	*0.73*<br>*0.92*	-	*0.53*<br>*0.85*	*0.69*<br>*0.94*	-	*0.50*<br>*0.81*	*0.65*<br>*0.90*
p value		***.07***	***.25***		***1.0***	***1.0***		***.035****	***.27***		***.51***	***1.0***		***< .0001****	***.73***		***.15***	***1.0***		***< .0001****	***1.0***		***0.007****	***1.0***		***< .0001****	***.77***		***.039****	***1.0***		***.004****	***.77***
**Observer 3**																																	
TP	21	14	21	14	14	14	51	34	45	26	23	24	82	54	75	35	26	29	94	58	85	44	31	40	78	50	70	27	16	23	35	21	28
ICC*	-	.75	.92	-	-	1.0	-	.62	.78	-	.80	.93	-	.59	.85	-	.75	.84	-	.50	.75	-	.75	.88	-	.61	.81	-	.66	.84	-	.65	.73
95% CI	-	*0.58*<br>*0.91*	*0.83*<br>*1.0*	-	-	-	-	*0.49*<br>*0.76*	*0.68*<br>*0.89*	-	*0.67*<br>*0.92*	*0.85*<br>*1.0*	-	*0.47*<br>*0.71*	*0.76*<br>*0.94*	-	*0.63*<br>*0.89*	*0.73*<br>*0.95*	-	*0.38*<br>*0.62*	*0.63*<br>*0.87*	-	*0.63*<br>*0.87*	*0.80*<br>*0.97*	-	*0.49*<br>*0.73*	*0.71*<br>*0.91*	-	*0.49*<br>*0.83*	*0.72*<br>*0.95*	-	*0.50*<br>*0.81*	*0.60*<br>*0.86*
p value		***.07***	***.25***		***1.0***	***1.0***		***.035****	***.79***		***.51***	***1.0***		***< .0001****	***.34***		***.15***	***.29***		***< .0001****	***.61***		***.002****	***1.0***		***< .0001****	***.58***		***.023****	***1.0***		***.004****	***1.0***

**ICC ****	-	1.0	1.0	-	1.0	1.0	-	.93	.97	-	.96	.97	-	.97	.98	-	.97	.96	-	.95	.97	-	.95	1.0	-	.98	.98	-	.96	.98	-	.95	.98

Note: FRACTURE, fast field echo resembling a CT using restricted echo-spacing. P_1, conventional MRI; P_2, FRACTURE sequence; TP, true positive; ICC*, intraclass correlation coefficient; CI, confidence interval; ICC**, inter-observer agreement. The *p*-values were statistically analyzed by McNemar test. *****, $p<0.05$.
